# Supplementary material for: Direct-Coupling Analysis of nucleotide coevolution facilitates RNA secondary and tertiary structure prediction
Source: Nucleic Acids Res. 2015 Sep 29;43(21):10444–55. doi: 10.1093/nar/gkv932 (PMC4666395; doi:10.1093/nar/gkv932)
Supplement: SUPPLEMENTARY DATA [file supp_43_21_10444__index.html]

Direct-Coupling Analysis of nucleotide coevolution facilitates RNA secondary and tertiary structure prediction — SUPPLEMENTARY DATA 

# Direct-Coupling Analysis of nucleotide coevolution facilitates RNA secondary and tertiary structure prediction

## SUPPLEMENTARY DATA

- SUPPLEMENTARY DATA
- SUPPLEMENTARY DATA
