# Supplementary material for: Direct-Coupling Analysis of nucleotide coevolution facilitates RNA secondary and tertiary structure prediction
Source: Nucleic Acids Res. 2015 Sep 29;43(21):10444–55. doi: 10.1093/nar/gkv932 (PMC4666395; doi:10.1093/nar/gkv932)
Supplement: SUPPLEMENTARY DATA [file supp_gkv932_SupplementRNADCA.pdf]

ONLINE ONLY: SUPPLEMENTAL INFORMATION

Direct-Coupling Analysis of nucleotide coevolution facilitates RNA secondary and tertiary structure prediction

E. De Leonardis, B. Lutz, S. Ratz, S. Cocco, R. Monasson, A. Schug, M. Weigt

Name of Riboswitch	PDB	Resolution PDB (Å)	RFAM Code	(effective) Number sequences in RFAM	PDB sequence length	RFAM sequence length*
Adenine	1y26	2.1	RF00167	2427 (588.88)	71	102
TPP	2gdi	2.05	RF00059	11197 (3347.90)	80	104
SAM-I	2gis	2.9	RF00162	4757 (1165.56)	94	107
c-di-GMP	3irw	2.7	RF01051	1990 (983.21)	98	87
Glycine	3owi	2.85	RF00504	6875 (1940.98)	88	90
Fluoride	3vrs	2.6	R01734	1267 (532.03)	52	64

Table S1 - Selected riboswitch families with Rfam and PDB information.

**Sequences and Consensus Secondary Structure of Riboswitches**
**1y26**<br>cgcuucauauaauccuaaugauaugguuugggaguuucuaccaagagccuuaaacucuugauuaugaagug<br>(((((((((...((((((.........))))))........((((((.......))))))..)))))))))
**2gdi**<br>ggacucggggugcccuucugcgugaaggcugagaaauacccguaucaccugaucuggauaaugccagcguagggaaguuc<br>(((((((((..(((............)))..........)))).....((((..((((......))))..))))..)))))
**2gis**<br>ggcuuaucaagagagguggagggacuggcccgaugaaacccggcaaccagaaauggugccaauuccugcagcggaaacguugaaagaugagcca<br>((((((((......(((...(((.....)))......))).(((.(((......))))))........(((((....)))))...)))))))).
**3irw**<br>gucacgcacagggcaaaccauucgaaagagugggacgcaaagccuccggccuaaaccauugcacuccgguagguagcggguuaccgaug<br>..((((......((...((((((....))))))...))..((((.((((((....((.........))..)))..)))))))..)).))
**3owi**<br>ggcucuggagagaaccguuuaaucggucgccgaaggagcaagcucugcggaaacgcagagugaaacucucaggcaaaaggacagaguc<br>(((((((......((((......)))).(((((..(((...........................))))).)))......)))))))
**3vrs**<br>gggcgaugaggcccgcccaaacugcccugaaaagggcugauggccucuacug<br>.......((((((..........((((......))))....)))))).....

Table S2 - Rfam consensus structure adapted to the structure-prediction target sequences

Direct-Coupling Analysis

In the following we briefly recall the main aspects of DCA, for a more detailed description containing technical details cf. Morcos et al.[1].

The aim of DCA is to fit a global statistical model, given under the form of a Potts model (or, equivalently, a pairwise Markov Random Field), such that the occurrence counts for individual residues and residue pairs are matched. While the underlying procedure assumes the availability of a good i.i.d. sample, the biological data, i.e. our input MSA of homologous RNA sequences, has three major problems: *(i)* Many columns contain a large fraction of gaps, and thus do not correspond to well-identifiable homology relations between different sequences. *(ii)* The sample is biased due to phylogeny and biased selection of sequenced species. *(iii)* The sample is relatively small, resulting in strong finite-sample artifacts. To decrease their impact, both effects are corrected for by some simple heuristic procedure:

(i) Gap pruning:
First of all we remove gapped columns from the alignment showing more than 50% of gaps. Within this procedure, we take care not to remove single residues included in the Rfam consensus secondary structure. Note that the final outcome is pretty insensitive to the exact threshold for gap removal, since more than 90% of all columns in the considered Rfam alignments have either less than 10% or more than 90% gaps.

(ii) Reweighting:
To decrease biases due to phylogenetic relations among sequences and due to the biased selection of species chosen for sequencing, we use a simple reweighting procedure for sequences. This procedure gives a lower statistical weight to sequences, which are too similar to be considered as statistically independent. To this aim we set a similarity threshold $0 < x < 1$, which has been empirically fixed to 0.9.
For each sequence $A^a = ({A_1}^a, {A_2}^a, \dots, {A_L}^a)$ in the MSA, we count the number m_a of sequences $A^b = \left({A_1}^b, {A_2}^b, \dots, {A_L}^b\right)$ whose sequence identity (seqid) with A^a is larger than xL (A^a itself being counted, too)

EQ.1

$$m_a = \left|\left\{b \mid 1 \le b \le M, seqid\left(A^a, A^b\right) = xL\right\}\right| .$$

In this context, sequence identity may be determined counting or not the gapped positions, final results are robust and do not depend systematically on the precise procedure. Here we chose to treat gaps equivalently to nucleotides, in agreement with later steps of DCA modeling.
Finally, the weight of a sequence is set to $1/m_a$, thus frequency counts result to be

EQ. 2

---

[1] Morcos, Faruck et al. "Direct-coupling analysis of residue coevolution captures native contacts across many protein families." *Proceedings of the National Academy of Sciences* 108.49 (2011): E1293-E1301.

$$f_i(A) = \frac{1}{M_{eff}} \sum_{a=1}^{M} \frac{1}{m_a} \delta_{A,A_I{}^a}, \qquad f_{ij}(A,B) = \frac{1}{M_{eff}} \sum_{a=1}^{M} \frac{1}{m_a} \delta_{A,A_i{}^a} \delta_{B,A_j{}^a},$$

with the effective number of sequences given by

EQ. 3

$$M_{eff} = \sum_{a=1}^{M} \frac{1}{m_a}.$$

(iii) Pseudocounts:

In order to correct for finite sample effects (in particular we need the covariance matrix to be invertible) we regularize $f_i(A)$and $f_{ij}(A,B)$ with pseudocounts

EQ. 4

$$\widehat{f_i}(A) = (1-\theta) f_i(A) + \frac{\theta}{5}, \qquad \widehat{f_{ij}}(A,B) = (1-\theta) f_{ij}(A,B) + \frac{\theta}{25}(1-\delta_{ij}) + \frac{\theta}{5}\delta_{ij}\delta_{AB}.$$

We fix $\theta = 0.5$, in accordance with Morcos et al..

Estimating coupling parameters:

Based on these sampling-corrected frequency counts, we compute the empirical covariance matrix C^{emp}

EQ. 5

$$C^{emp}{}_{ij}(A,B) = \hat{f}_{ij}(A,B) - \hat{f}_i(A)\, \hat{f}_j(B).$$

Given that only 4 out of the 5 symbols {A,C,G,U,-} are effectively independent, the full correlation matrix C^{emp} of size 5*L*x5*L* has *L* null modes. Without loss of generality (the mathematical justification is based on a reparameterization invariance of the Potts model and described in detail in Morcos et al.) we restrict the covariance matrix to the full-rank 4*L*x4*L* submatrix $\hat{C}^{emp}$ containing only *A,B*={A,C,G,U}, but not the gap symbol "-". According to the mean-field solution of the inverse Potts model, couplings between nucleotides can be estimated by the inverse of the reduced covariance matrix $\hat{C}^{emp}$,

EQ. 6

$$e_{ij}(A,B) \simeq -\left(\left(\hat{C}^{emp}\right)^{-1}\right)_{ij}(A,B),$$

whereas gap-gap and gap-nucleotide couplings are set to zero, $e_{ij}(A,-) = e_{ij}(-,A) = 0$.

Ranking residue pairs:

In order to rank residue pairs, we define a scalar coupling score (called Fapc) by calculating first the Frobenius norm for each pair,

EQ. 7

$$F_{ij} = \sqrt{\sum_A \sum_B \left|\hat{e}_{ij}(A,B)\right|^2} \ ,$$

where $\hat{e}_{ij}$is the coupling matrix transformed such as the value of F_{ij} is minimized with respect to the reparametrization symmetry (i.e. in between all equivalent parametrizations we use the one minimizing the Frobenius norm of the couplings):

EQ. 8

$$\widehat{e_{ij}}(A,B) = e_{ij}(A,B) - < e_{ij}(A,B) >_A - < e_{ij}(A,B) >_B + < e_{ij}(A,B) >_{AB}.$$

In a last step, we add the average-product correction (APC)

EQ. 9

$$(F^{apc})_{ij} = F_{ij} - APC_{ij} = F_{ij} - \frac{<F_{ij}>_i <F_{ij}>_j}{<F_{ij}>_{ij}} \ ,$$

which was empirically found to improve contact prediction.

Generalized Nussinov algorithm: The dependence on the number of coevolution measures included into the scoring matrix

Over-pairing is a well-known issue of the generalized Nussinov algorithm in case of non-negative scores such as MI. Moreover, even though scores corrected with APC include some negative elements, they do not prevent this error. An alternative construction for the Nussinov score matrix is needed. To solve this problem we use some information coming from the sequence we want to fold: We initialize the score matrix with 0 for all possible W-C base pairs and -1 for all other pairs. Then we substitute the corresponding zero entries with some of the highest coevolution measures. The number of considered highest scores is n*L, L being the length of the sequence. In Figure SI2 we study the sensitivity and the precision of the predicted secondary structure in function of this parameter. As one can see, the behavior shown in Fig 2 in the main text is qualitatively conserved for all values of n: DCA outperforms MI and MIapc in sensitivity while they behave quite similarly when the precision is concerned. Moreover note that for fixed sensitivity, DCA shows an higher precision compared to MI scores. We fix the threshold to n=1 comparing our results with those of the Rfam consensus secondary structure: This choice represents the point where the generalized Nussinov with DCA reaches the quality of the consensus secondary structure.

Structure Prediction

We closely follow the procedures from Kladwang et al. [2] in Rosetta:

---

[2] Kladwang, Wipapat et al. "A two-dimensional mutate-and-map strategy for non-coding RNA structure." *Nature chemistry* 3.12 (2011): 954-962.

setup_rna_assembly_jobs.py

First, ideal A-helices are created based on the secondary structure information via, for the example 1y26,

```
rna_helix.exe  -fasta stem1_1y26.fasta -out:file:silent stem1_1y26.out
```

with stem1_1y26.fast containing the sequence of the P1-helix from 1y26 as determined by the consensus secondary structure in RFAM.:

```
>stem1_1y26.fasta
cgcuucauauaugaagug
```

In a second step RNA junctions and loop motifs are created:

```
rna_denovo.exe  -fasta motif1_1y26.fasta -params_file motif1_1y26.params -
nstruct 4000 -out:file:silent motif1_1y26.out -cycles 5000 -mute all -
close_loops -close_loops_after_each_move -minimize_rna -
in:file:silent_struct_type rna -in:file:silent  stem1_1y26.out stem2_1y26.out
stem3_1y26.out -chunk_res   1-9 47-55 13-24 33-44
```

Last, motifs and helices are combined into full models while considering tertiary constraints:

```
rna_denovo.exe -constant_seed -jran 1 -minimize_rna -fasta 1y26.fasta -
in:file:silent_struct_type binary_rna -cycles 20000 -nstruct 50000 -
out:file:silent 1y26_100TpFp.out -params_file 1y26_assemble.params -cst_file
tertiary_constraint.cst -close_loops  -in:file:silent  stem1_1y26.out
stem2_1y26.out stem3_1y26.out motif1_1y26.out motif2_1y26.out motif3_1y26.out
-chunk_res  1-9 63-71 13-18 28-33 42-47 55-60 1-18 28-47 55-71 13-33 42-60
```

Tertiary constraints are included via the file tertiary_constraint.cst:

```
[ atompairs ]
N1 23 C6 52 FADE -100 26 20 -2 2
N1 23 N1 52 FADE -100 26 20 -2 2
N1 23 C2 52 FADE -100 26 20 -2 2
N1 23 N3 52 FADE -100 26 20 -2 2
C2 23 C2 52 FADE -100 26 20 -2 2
[...]
```

For each of the 6 sets of predictions for each riboswitch (no tertiary constraints, 25/100 MIapc, 25/100 DCA, full contact map, cf. main text) we run 12 single-core simulations with different

random seeds for 3 days each on the HPC resources of the bwUniCluster at KIT[3]. After that time the jobs are cancelled, resulting in the same CPU time spent on each prediction and in about 2000-6000 models.

Mapping of residue contacts to atomic contacts

Residue-residue contacts need to be mapped onto a set of atom-atom contacts. To this end, we choose a set of characteristic RNA structures proposed by the group of Eric Westhof[4]. This set classifies possible inter-nucleotide contacts by their relative base coordination and provides a collection of representative structures to determine typical atom-atom distances. For a given nucleotide-nucleotide contact predicted by DCA, all representative structures are analyzed and the averages of according atom-atom distances are calculated. Atom-atom contacts with mean values less than 6 Å and standard deviations less than 3 Å are then included in the model.

Long Range vs. Short Range Contacts

Prior work adds residue-residue tertiary constraints as short-range constraints in ROSETTA v3.5 [5]. To increase the accuracy of the prediction, we introduce atomic constraints between specific atoms of the two nucleic acids forming a contact (see above). As implementation, we compare a “short-ranged contact” implementation similar to the prior work (FADE -100 10 2 -2 2) with a new implementation as “long-ranged contacts” (FADE -100 26 20 -2 2). Respectively, these constraints provide a energetic bonus of 2kcal/mol for the constrained atoms if they are closer than 8 (6) Å. This energetic bonus fades to zero for distances beyond 10(26) Å by a cubic spline. We find that the new long-range potential results in higher quality predictions (old data not shown). We attribute this to the provided long-ranged gradient which helps the Monte-Carlo optimization procedure in Rosetta fulfilling the constraints as well as being more forgiving of false positives.

Clustering of Rosetta Results

All structure predictions from each riboswitch prediction are ranked by their score. The lowest score is the reference conformation of the first cluster. Then subsequently higher-score conformations are compared to all existing clusters by their heavy-atom RMSD. In case they are within a threshold of 4Å to any existing cluster, they are associated with this cluster, otherwise this conformation forms the reference structure of a new cluster. Each entry in table 1 lists the lowest RMSD of the first, first 5, or first 10 reference cluster conformations to the native state.

---

[3] 16-way Intel Xeon compute nodes with two Octa-core Intel Xeon processors E5-2670 (Sandy Bridge) with a clock speed of 2.6 GHz, 8x256 KB of level 2 cache and 20 MB level 3 cache. Each node has 64 GB of main memory, local disks with 2 TB and an adapter to connect to the InfiniBand 4X FDR interconnect.

[4] Leontis, Neocles B, Jesse Stombaugh, and Eric Westhof. "The non Watson–Crick base pairs and their associated isostericity matrices." *Nucleic acids research* 30.16 (2002): 3497-3531.

[5] Kladwang, Wipapat et al. "A two-dimensional mutate-and-map strategy for non-coding RNA structure." *Nature chemistry* 3.12 (2011): 954-962.

Relation between Rosetta results and DCA predictions

Even if it is shown that DCA can help Rosetta to find a good prediction when compared to the native structure (cf. Table 1 in the main text), results can strongly depend on the riboswitch family considered. In the following we try to understand *a posteriori*, which elements in the inference have contributed to the RMSD result obtained.

We consider separately the six families referring to Figs. SI4 and SI6:

RF00059 2gdi

TP rates for MIapc and DCA show high performances in both the cases, however in this case DCA has some crucial TP predictions that are not there when MIapc is used (in particular when 100 predictions are used): sites 30-71, sites 38-59, sites 12-61. By adding these pairs we force Rosetta to reproduce the corresponding clusters of contacts obtaining a much better structural prediction. Indeed this is a clear example of how a "small" improvement in the inference step results in a higher improvement at the structure level.

RF00504 3owi

Looking at the contact map of this family, we notice that the secondary structure is predominant over the whole tertiary structure: There are only three main clusters of contacts sufficiently far away from the secondary structure and from the backbone (long-range). Moreover none of these clusters are properly predicted by MIapc or DCA (only one TP is found in the +100 prediction DCA list), thus both the inference methods poorly perform within Rosetta compared to secondary-structure only predictions.

RF00162 2gis

Even if the TP rates of this family are quite good, results in term of RMSD show a great variability, both for MIapc and for DCA. Again, looking at predicted contact maps (Fig. SI6) we can argue that the pretty large number of FPs found far from native contacts, can give rise to competing structures within Rosetta predictions.

RF00167 1y26

For this family the hypothesis of competing structures can explain why DCA predictions obtain very good results when only 25 predictions are used instead of 100. The latter actually include two big clusters of FP: Rosetta cannot produce a structure that satisfies both the TP and the FP at the same time, thus two classes of structures coexist. Eventually, given such a strong coevolution signal, we cannot exclude that the "wrong" class actually is a different conformation of this riboswitch.

R01051 3irw

In this case, we note that MIapc performs similarly with 25 or 100 predictions, while DCA is slightly better with 100. Both outperform secondary-structure only predictions. TP rates show that the quality of the inference is in any case quite good, the main difference we can see regards the cluster of contacts in correspondence to sites 25-70: it is reached by MIapc (both +25 and +100) and by DCA +100. In the only case, in which it is not found (DCA +25), RMSD values are significantly higher. One can thus argue that this contact cluster carries very important information for a correct fold prediction.

RF01734 3vrs

The coevolution signal is very noisy and thus DCA and MIapc cannot help folding compared to secondary-structure alone. However, the results are quite good if compared to what Rosetta can obtain when the whole contact map of this RS is given. The latter case fails since Rosetta consistently scores higher RMSD models better than lower RMSD ones, cf. Fig. SI7. This indicates a general problem – the quality of model scores – which clearly goes beyond this work.

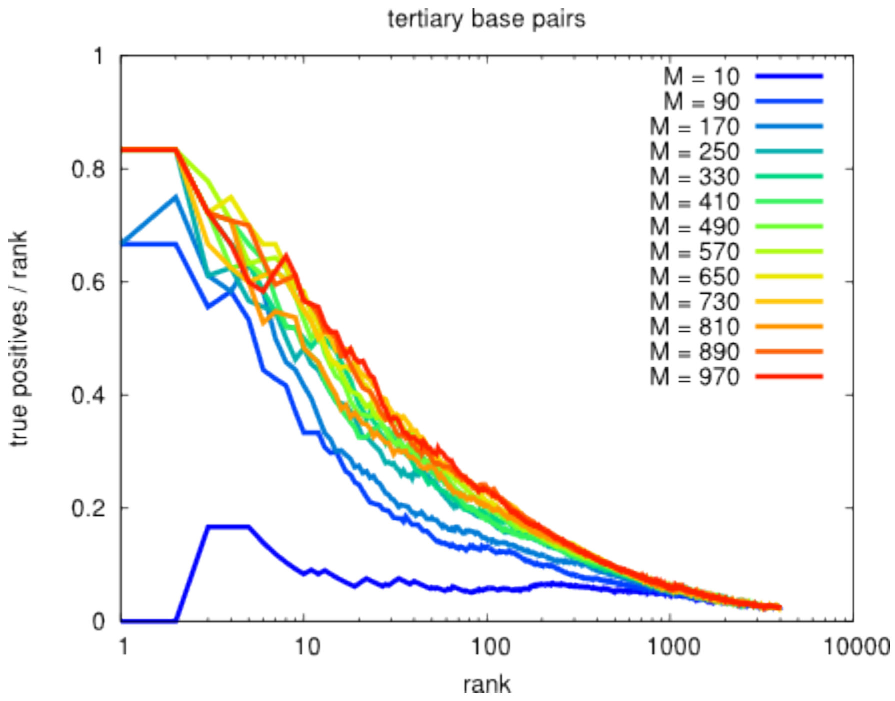

Figure SI1

Sub-alignment analysis. Each line represents the averaged TP rate (tertiary-contact prediction with a 8Å cutoff) for the 6 families obtained with a randomly chosen sub-alignment of M sequences, for different values of M. The TP rates have to be compared to panel D in Fig 4 in the main text.

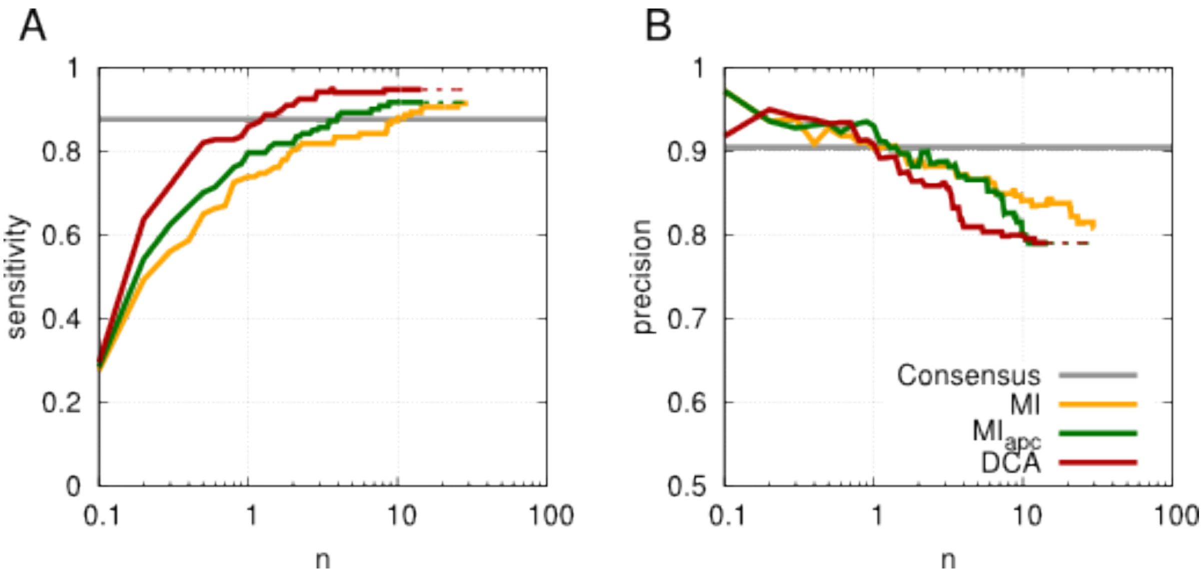

Figure SI2

Sensitivity TP/(TP+FN) **(A)** and precision TP/(TP+FP) **(B)** for the generalized Nussinov predictions for different value of n, with n*L being the number of coevolution scores inserted in the Nussinov scoring matrix, and L being the sequence length. Colored lines show results for DCA (red), MI (yellow) and MIapc (green), while the grey straight lines show the sensitivity and the precision of the Rfam consensus secondary structure. The dotted lines represent n values for which an average over the 6 families is no longer possible due to size differences.

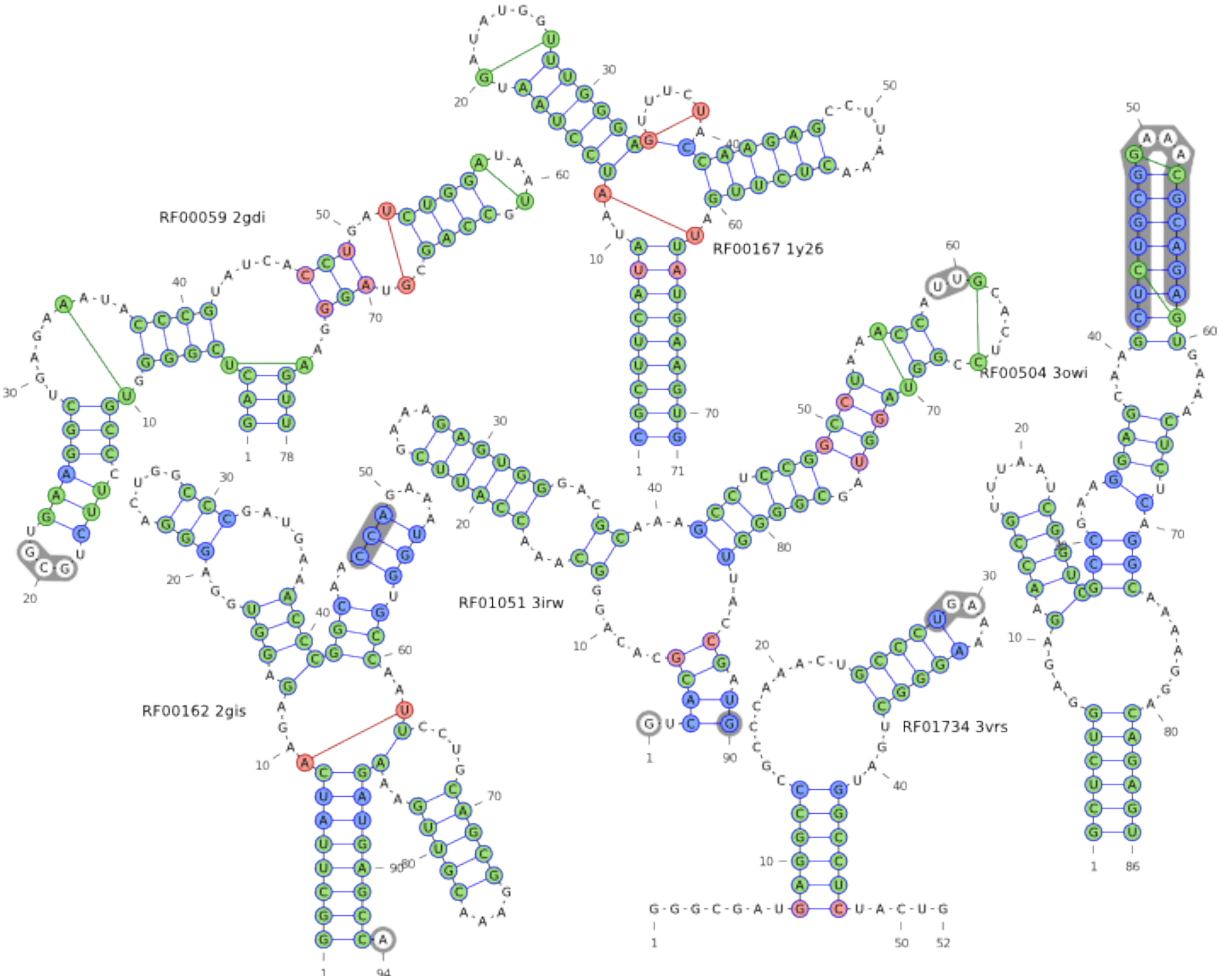

Figure SI3

Comparison between PDB secondary structures and predictions using MIapc and DCA (cf. Fig 3 main text). The underlying secondary structure (blue lines) is derived from base pairs in the PDB file. Red-filled base pairs belong exclusively to the DCA predicted structures (DCA TP), yellow-filled exclusively to the MIapc predicted structures (MIapc TP), and green-filled ones are found in both the DCA and the MIapc predictions (both DCA and MIapc TP). Blue-filled base pairs have not been predicted by coevolutionary analysis (both DCA and MIapc FN). Lines linking nucleotides outside the secondary structure represent false positives: red lines for DCA and green lines for both DCA and MI. Grey-shadowed bases represent non-aligned regions between PDB sequence and Rfam alignment. The list of WC base-pairs in the PDB files is extracted with RNAView package[6].

[6] Yang, Huanwang et al. "Tools for the automatic identification and classification of RNA base pairs." *Nucleic acids research* 31.13 (2003): 3450-3460.

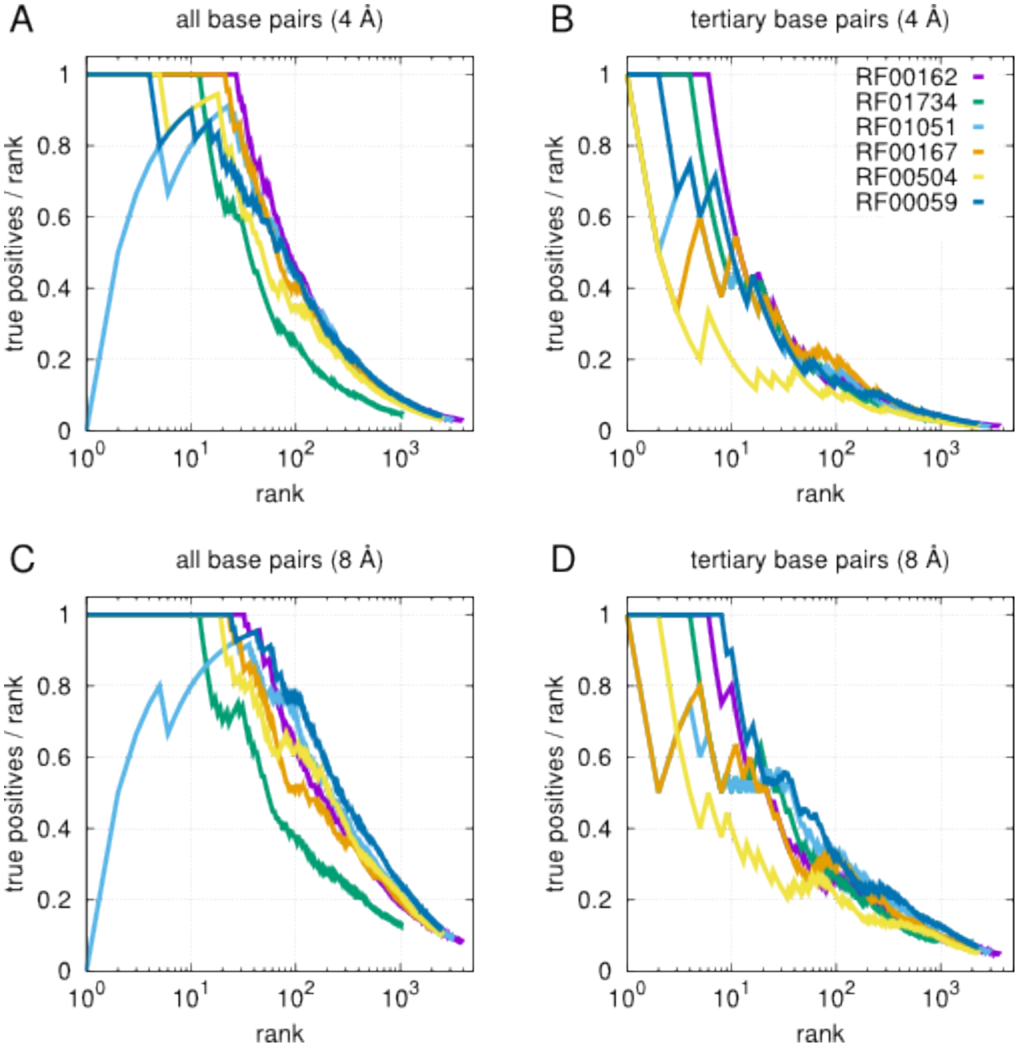

Figure SI4
Same as Fig. 4 in the main text but only DCA results for the 6 individual riboswitches are shown.

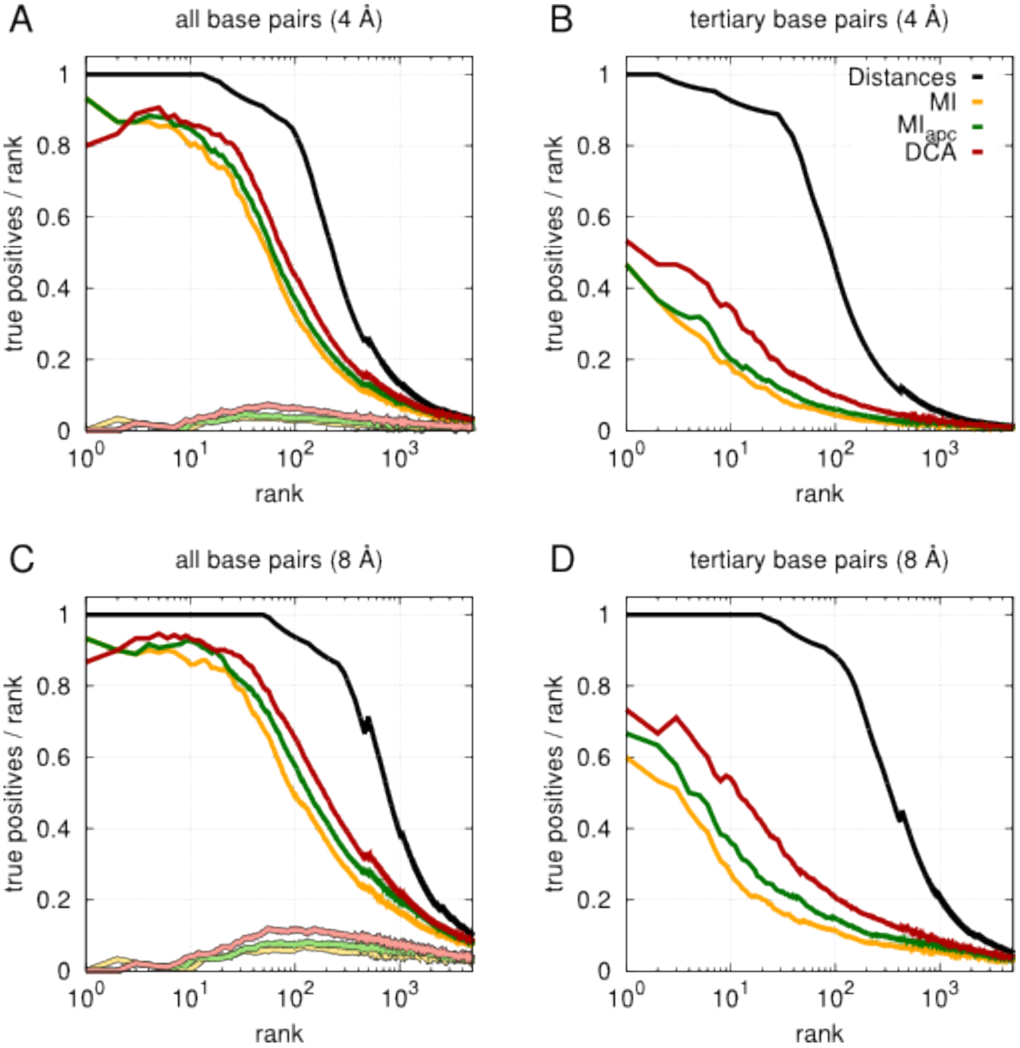

Figure SI5

Averaged TP rates on a larger set of RNA families having a corresponding complete PDB X-ray diffraction structure with less than 3Å resolution and an Rfam family with more than 1000 sequences. The complete list of families and structures is the following: RF00162 2gis, RF01734 3vrs, RF01051 3irw, RF00167 1y26, RF00504 3owi, RF00059 2gdi, RF00001 3cc2, RF00163 2oeu, RF00017 1l9a, RF00010 1u9s, RF00050 3f2q, RF02001 3bwp, RF00168 3dil, RF00380 2qbz, RF00023 4ab.

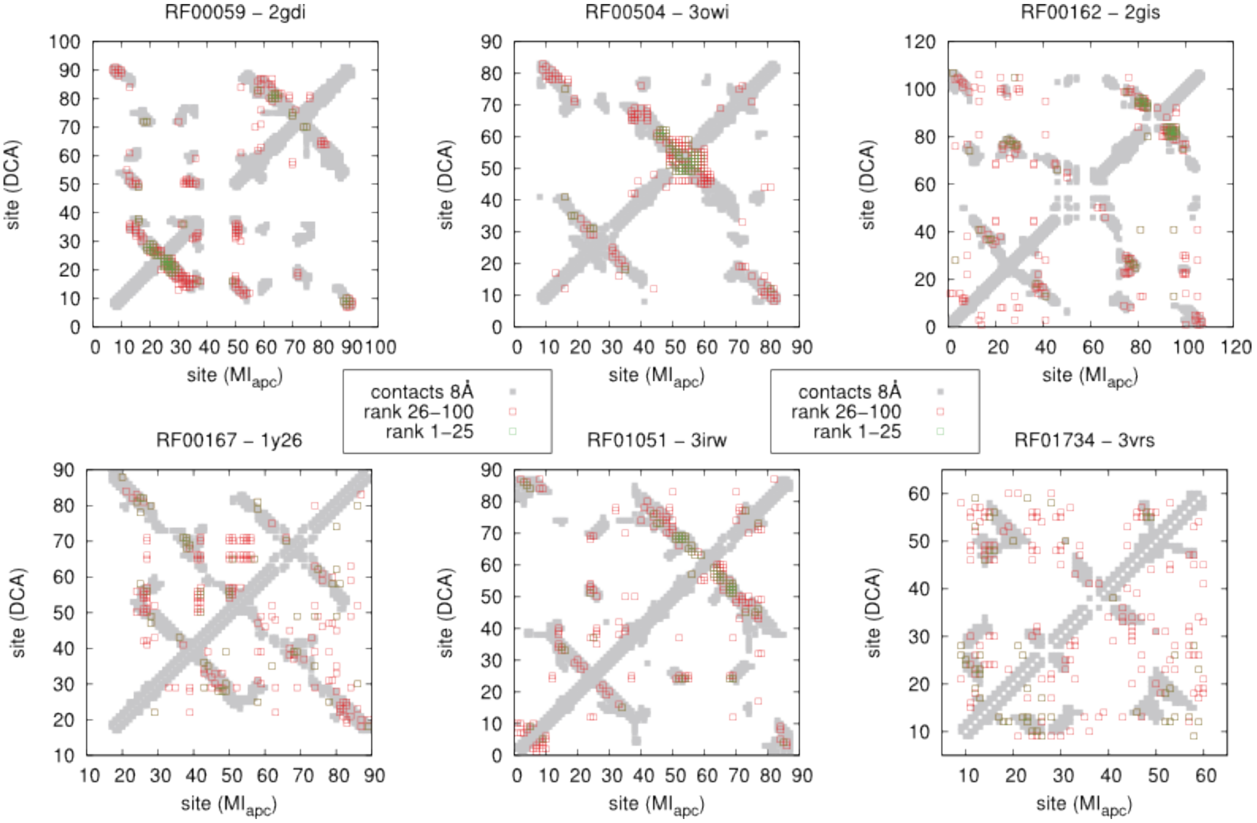

Figure SI6

Contact maps for the six riboswitches with predictions included as residue-residue tertiary constraints in Rosetta. Grey dots show the native structure where two sites are considered to be in contact if they are closer than 8Å. Green open squares represent the first 25 predictions and red open squares represent predictions from rank 26 to rank 100. We show in the top-left triangle the DCA predictions, while in the bottom-right one the MIapc predictions. Note that the quality of the inference both for DCA and MIapc is very well correlated with the ratio Meff/L shown in table 1 in the main text: PF00059 and PF00504 have Meff/L-values above 20, PF01051 and RF00162 slightly above 10, RF00167 and RF01734 below 10.

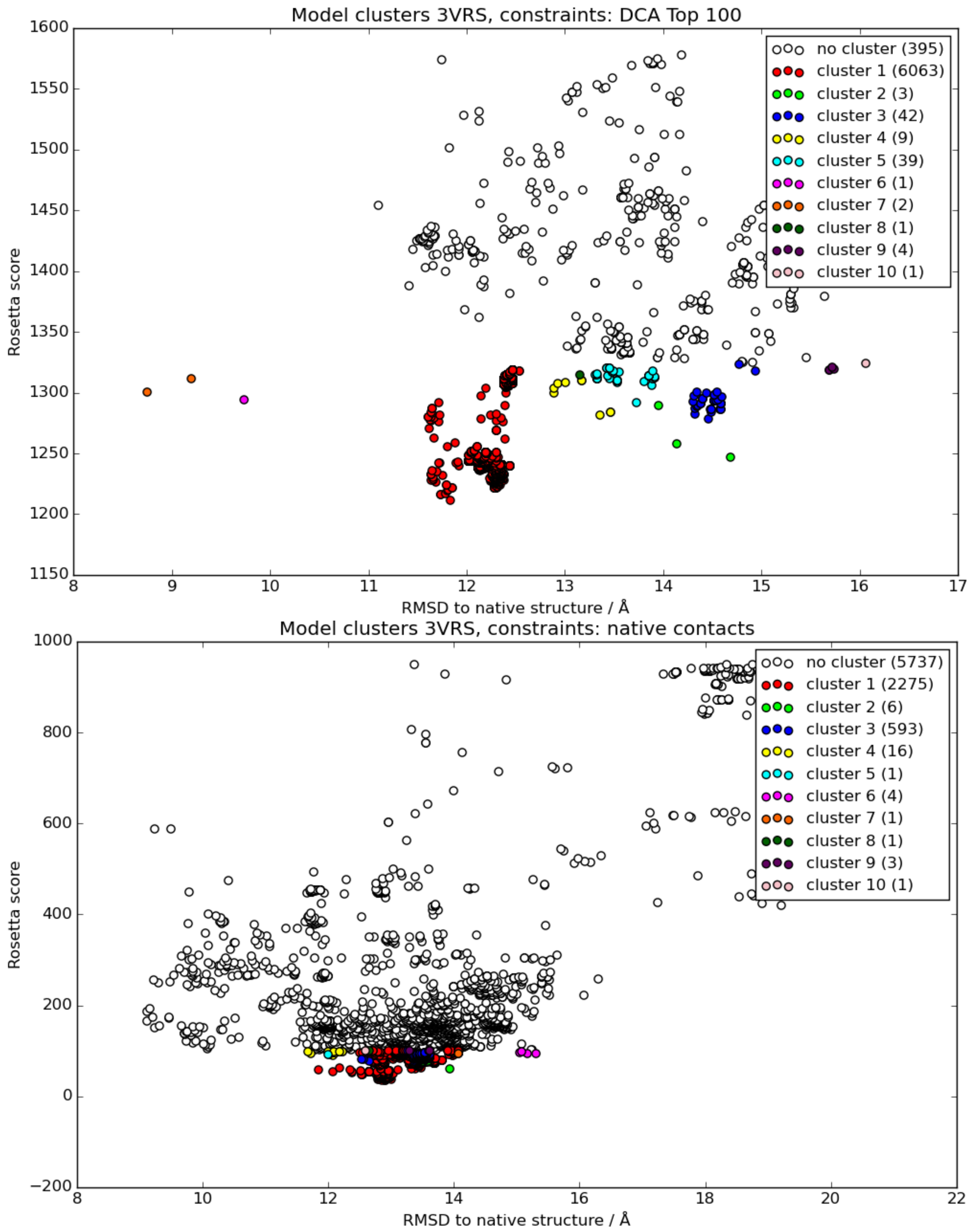

Figure SI7

Scatter plots of Rosetta predictions for 3vrs. RMSD vs. Rosetta score for the predictions using the top 100 DCA contacts (top) and all native contacts (bottom). While RMSDs lower than 10Å are more populated in the bottom case, they are not well detected by Rosetta's scoring system.

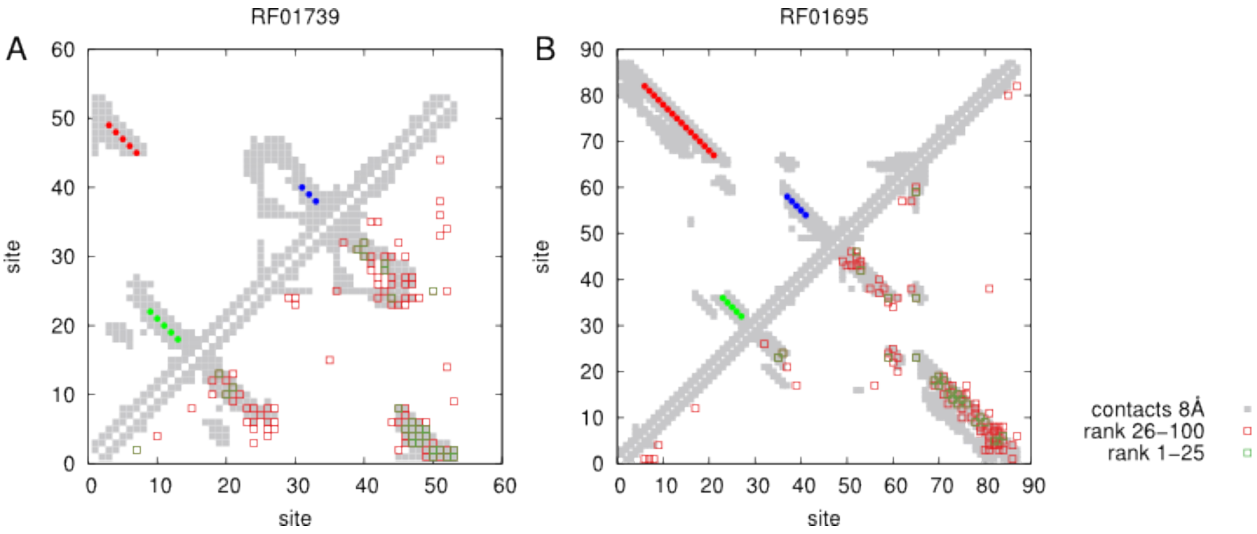

Figure SI8

Predicted contact maps of (A) glnA riboswitch and (B) C4 antisense RNA based on DCA the best-scoring Rosetta model. Upper-left triangle: the predicted secondary structure is shown. Colors refer to Fig. 6 in the main text. Lower-right triangle: DCA predictions. Green squares represent the top 25 predictions while red squares predictions from rank 26 to rank 100.
